# 2-[4-(Trifluoro­meth­oxy)phen­yl]-1*H*-benzimidazole

**DOI:** 10.1107/S1600536813001220

**Published:** 2013-01-19

**Authors:** Nikhath Fathima, M. S. Krishnamurthy, Noor Shahina Begum

**Affiliations:** a Department of Studies in Chemistry, Bangalore University, Bangalore 560 001, India

## Abstract

In the title compound, C_14_H_9_F_3_N_2_O, the best planes of the benzimidazole group and benzene ring form a dihedral angle of 26.68 (3)°. In the crystal, N—H⋯N hydrogen bonds link the mol­ecules into infinite chains parallel to the *c* axis. Stacking inter­actions between the benzimidazole groups [centroid–centroid distance = 3.594 (5) Å] assemble the mol­ecules into layers parallel to (100). The trifluoro­methyl group is disordered over three sets of sites with site-occupancy factors of 0.787 (4), 0.107 (7) and 0.106 (7).

## Related literature

For therapeutic and medicinal properties of benzimidazole derivatives, see: Chimirri *et al.* (1991 ▶); Benavides *et al.* (1995 ▶); Ishihara *et al.* (1994 ▶); Kubo *et al.* (1993 ▶). For related structures, see: Jian *et al.* (2006 ▶); Rashid, Tahir, Yusof *et al.* (2007 ▶); Rashid, Tahir, Kanwal *et al.* (2007 ▶).
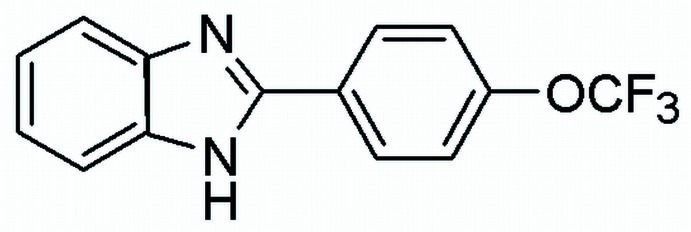


## Experimental

### 

#### Crystal data

C_14_H_9_F_3_N_2_O
*M* *_r_* = 278.23
Monoclinic, 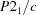

*a* = 14.476 (6) Å
*b* = 9.312 (4) Å
*c* = 9.835 (4) Å
β = 108.192 (8)°
*V* = 1259.5 (9) Å^3^
*Z* = 4
Mo *K*α radiation
μ = 0.13 mm^−1^
*T* = 296 K
0.18 × 0.16 × 0.16 mm

#### Data collection

Bruker SMART APEX CCD detector diffractometer
Absorption correction: multi-scan (*SADABS*; Bruker, 1998 ▶) *T*_min_ = 0.978, *T*_max_ = 0.980
6284 measured reflections
2209 independent reflections
1333 reflections with *I* > 2σ(*I*)
*R*_int_ = 0.077

#### Refinement

*R*[*F* ^2^ > 2σ(*F* ^2^)] = 0.064
*wR*(*F* ^2^) = 0.157
*S* = 1.00
2209 reflections
210 parameters
21 restraints
H-atom parameters constrained
Δρ_max_ = 0.26 e Å^−3^
Δρ_min_ = −0.22 e Å^−3^

### 

Data collection: *SMART* (Bruker, 1998 ▶); cell refinement: *SAINT-Plus* (Bruker, 1998 ▶); data reduction: *SAINT-Plus*; program(s) used to solve structure: *SHELXS97* (Sheldrick, 2008 ▶); program(s) used to refine structure: *SHELXL97* (Sheldrick, 2008 ▶); molecular graphics: *ORTEP-3 for Windows* (Farrugia, 2012 ▶) and *CAMERON* (Watkin *et al.*, 1996 ▶); software used to prepare material for publication: *WinGX* (Farrugia, 2012 ▶).

## Supplementary Material

Crystal structure: contains datablock(s) global, I. DOI: 10.1107/S1600536813001220/gk2550sup1.cif

Structure factors: contains datablock(s) I. DOI: 10.1107/S1600536813001220/gk2550Isup2.hkl

Supplementary material file. DOI: 10.1107/S1600536813001220/gk2550Isup3.cml

Additional supplementary materials:  crystallographic information; 3D view; checkCIF report

## Figures and Tables

**Table 1:** Hydrogen-bond geometry (Å, °)

*D*—H⋯*A*	*D*—H	H⋯*A*	*D*⋯*A*	*D*—H⋯*A*
N2—H21⋯N1^i^	0.86	2.07	2.864 (4)	154

Symmetry code: (i) 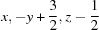.

**Fig. 1.**
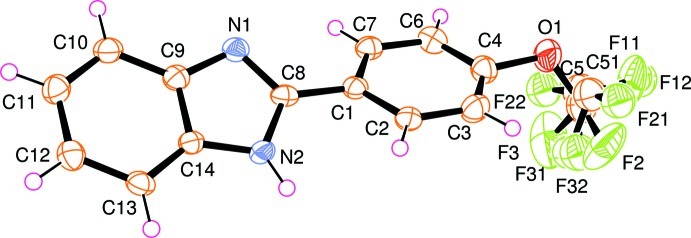
The molecular structure of the title compound with the atom numbering scheme. Displacement ellipsoids are drawn at the 50% probability level. H atoms are presented as small spheres of arbitrary radius.The trifluoromethyl group is disordered over three sites.

**Fig. 2.**
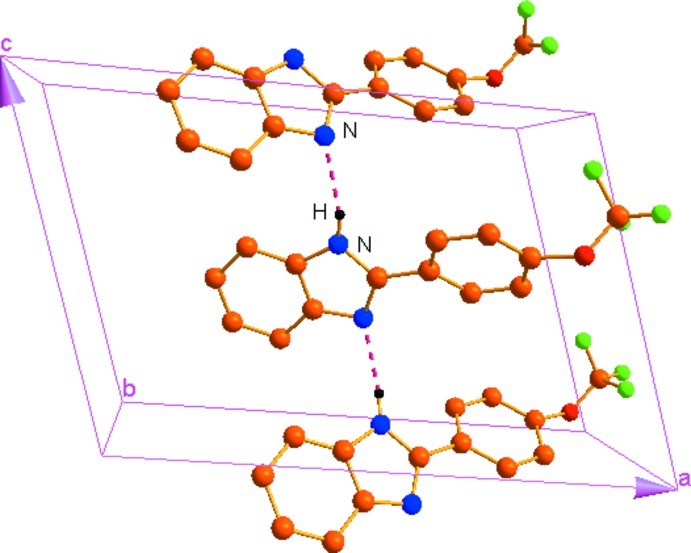
A view of the intermolecular hydrogen bonds(dotted lines) in the crystal structure of the title compound. H atoms non participating in H-bonding and minor components of the disordered CF3 group were omitted for clarity.

## supplementary crystallographic information

### Comment

Benzimidazole and its derivatives are known to exhibit a wide variety of pharmacological activities such as anti-HIV (Chimirri *et al.*, 1991), antihistaminic (Benavides *et al.*, 1995), antiulcer (Ishihara *et al.*, 1994), antihypertensive (Kubo *et al.*, 1993). The benzimidazole and phenyl groups form a dihedral angle of 26.68 (3)°. The bond lengths and angles of the benzimidazole moiety in the title compound are in good agreement with those observed in other benzimidazole derivatives (Jian *et al.*, 2006; Rashid, Tahir, Yusof *et al.*, 2007; Rashid, Tahir, Kanwal *et al.*, 2007). The N1—C8 and N2—C8 distances were found to be 1.338 (5) Å and 1.356 (5) Å, respectively. The trifluoromethyl group is disordered over three sites with occupancy factors 0.787 (4), 0.107 (7) and 0.106 (7). The molecules are linked by intermolecular N—H···N hydrogen bonds to form infinite chains parallel to the *c* axis. Additionally, the crystal packing is further stabilized by π -π stacking interactions between the benzimidazole groups [interplanar distance 3.594 (5) Å].

### Experimental

A mixture of 4-(trifluoromethoxy)benzaldehyde (10 mmol, 0.19 g) and *o*-phenyldiamine (10 mmol, 0.19 g) in benzene (2 ml) was refluxed for 6 h on a water bath. The reaction mixture was cooled. The solid separated, was filtered and dried (yield: 0.26 g, 75% and m.p. 503–508 K). Pale yellow crystals of the title compound were obtained by slow evaporation from a solution in ethyl acetate.

### Refinement

All H atoms were included in calculated positions, with C—H bond distances of 0.93 Å and N—H = 0.86 Å and refined in a riding model approximation with *U*_iso_(H) = 1.2*U*_eq_(C,*N*). The trifluoromethyl group is disordered over three sites with the occupancy factors 0.787 (4), 0.107 (7) and 0.106 (7). The atoms of the minor components were refined isotropically with a common displacement parameter for each group. The geometry of the minor components was restrained to that of the major component with the SAME instruction of *SHELXL97* (Sheldrick, 2008).

### Crystal data

**Table:**

C_14_H_9_F_3_N_2_O	*F*(000) = 568
*M**_r_* = 278.23	*D*_x_ = 1.467 Mg m^−^^3^
Monoclinic, *P*2_1_/*c*	Mo *K*α radiation, λ = 0.71073 Å
Hall symbol: -P 2ybc	Cell parameters from 2209 reflections
*a* = 14.476 (6) Å	θ = 2.6–25.0°
*b* = 9.312 (4) Å	µ = 0.13 mm^−^^1^
*c* = 9.835 (4) Å	*T* = 296 K
β = 108.192 (8)°	Block, yellow
*V* = 1259.5 (9) Å^3^	0.18 × 0.16 × 0.16 mm
*Z* = 4	

### Data collection

**Table:**

Bruker SMART APEX CCD detector diffractometer	2209 independent reflections
Radiation source: fine-focus sealed tube	1333 reflections with *I* > 2σ(*I*)
Graphite monochromator	*R*_int_ = 0.077
ω scans	θ_max_ = 25.0°, θ_min_ = 2.6°
Absorption correction: multi-scan (*SADABS*; Bruker, 1998)	*h* = −17→15
*T*_min_ = 0.978, *T*_max_ = 0.980	*k* = −8→11
6284 measured reflections	*l* = −11→11

### Refinement

**Table:**

Refinement on *F*^2^	Primary atom site location: structure-invariant direct methods
Least-squares matrix: full	Secondary atom site location: difference Fourier map
*R*[*F*^2^ > 2σ(*F*^2^)] = 0.064	Hydrogen site location: inferred from neighbouring sites
*wR*(*F*^2^) = 0.157	H-atom parameters constrained
*S* = 1.00	*w* = 1/[σ^2^(*F*_o_^2^) + (0.0655*P*)^2^] where *P* = (*F*_o_^2^ + 2*F*_c_^2^)/3
2209 reflections	(Δ/σ)_max_ = 0.001
210 parameters	Δρ_max_ = 0.26 e Å^−^^3^
21 restraints	Δρ_min_ = −0.22 e Å^−^^3^

### Special details

**Table:**

Geometry. All s.u.'s (except the s.u. in the dihedral angle between two l.s. planes) are estimated using the full covariance matrix. The cell s.u.'s are taken into account individually in the estimation of s.u.'s in distances, angles and torsion angles; correlations between s.u.'s in cell parameters are only used when they are defined by crystal symmetry. An approximate (isotropic) treatment of cell s.u.'s is used for estimating s.u.'s involving l.s. planes.
Refinement. Refinement of *F*^2^ against ALL reflections. The weighted *R*-factor *wR* and goodness of fit *S* are based on *F*^2^, conventional *R*-factors *R* are based on *F*, with *F* set to zero for negative *F*^2^. The threshold expression of *F*^2^ > 2σ(*F*^2^) is used only for calculating *R*-factors(gt) *etc*. and is not relevant to the choice of reflections for refinement. *R*-factors based on *F*^2^ are statistically about twice as large as those based on *F*, and *R*- factors based on ALL data will be even larger.

### Fractional atomic coordinates and isotropic or equivalent isotropic displacement parameters (Å^2^)

**Table:**

	*x*	*y*	*z*	*U*_iso_*/*U*_eq_	Occ. (<1)
C1	0.3459 (2)	0.8348 (3)	0.4611 (3)	0.0246 (8)	
C2	0.3309 (2)	0.9562 (3)	0.3748 (3)	0.0297 (8)	
H2	0.3786	0.9857	0.3359	0.036*	
C3	0.2445 (2)	1.0344 (4)	0.3460 (4)	0.0361 (9)	
H3	0.2352	1.1177	0.2912	0.043*	
C4	0.1735 (2)	0.9856 (4)	0.4004 (4)	0.0333 (9)	
C6	0.1860 (2)	0.8644 (3)	0.4854 (3)	0.0321 (9)	
H6	0.1368	0.8332	0.5206	0.039*	
C7	0.2734 (2)	0.7900 (3)	0.5172 (3)	0.0284 (8)	
H7	0.2836	0.7097	0.5763	0.034*	
C8	0.4390 (2)	0.7578 (3)	0.4969 (3)	0.0245 (8)	
C9	0.5675 (2)	0.6361 (3)	0.6072 (3)	0.0260 (8)	
C10	0.6411 (2)	0.5565 (3)	0.7050 (3)	0.0316 (9)	
H10	0.6339	0.5228	0.7901	0.038*	
C11	0.7245 (2)	0.5305 (3)	0.6695 (4)	0.0327 (9)	
H11	0.7743	0.4778	0.7323	0.039*	
C12	0.7367 (2)	0.5806 (3)	0.5422 (3)	0.0311 (8)	
H12	0.7946	0.5624	0.5233	0.037*	
C13	0.6639 (2)	0.6567 (3)	0.4443 (3)	0.0277 (8)	
H13	0.6709	0.6883	0.3583	0.033*	
C14	0.5804 (2)	0.6840 (3)	0.4794 (3)	0.0233 (8)	
O1	0.08661 (17)	1.0648 (3)	0.3804 (3)	0.0460 (7)	
N1	0.47847 (18)	0.6843 (3)	0.6176 (3)	0.0272 (7)	
N2	0.49671 (18)	0.7606 (3)	0.4113 (3)	0.0260 (7)	
H21	0.4836	0.8024	0.3295	0.031*	
C5	0.0271 (4)	1.0787 (7)	0.2470 (6)	0.0534 (16)	0.787 (4)
F1	−0.0537 (4)	1.1368 (8)	0.2540 (9)	0.0740 (15)	0.787 (4)
F2	0.0616 (3)	1.1639 (8)	0.1658 (6)	0.0954 (17)	0.787 (4)
F3	0.0071 (4)	0.9548 (6)	0.1788 (7)	0.110 (2)	0.787 (4)
C51	0.0312 (14)	1.109 (2)	0.268 (3)	0.047 (6)*	0.107 (7)
F11	−0.0580 (16)	1.145 (3)	0.259 (7)	0.047 (6)*	0.107 (7)
F21	0.0781 (14)	1.2304 (19)	0.261 (3)	0.047 (6)*	0.107 (7)
F31	0.030 (3)	1.033 (3)	0.154 (4)	0.047 (6)*	0.107 (7)
C52	0.010 (2)	1.062 (3)	0.270 (3)	0.056 (7)*	0.106 (7)
F12	−0.066 (4)	1.144 (3)	0.251 (8)	0.056 (7)*	0.106 (7)
F22	−0.0187 (16)	0.928 (2)	0.282 (3)	0.056 (7)*	0.106 (7)
F32	0.037 (4)	1.064 (4)	0.154 (4)	0.056 (7)*	0.106 (7)

### Atomic displacement parameters (Å^2^)

**Table:**

	*U*^11^	*U*^22^	*U*^33^	*U*^12^	*U*^13^	*U*^23^
C1	0.0277 (18)	0.0240 (18)	0.0207 (17)	−0.0013 (14)	0.0055 (14)	−0.0033 (14)
C2	0.031 (2)	0.037 (2)	0.0256 (18)	0.0045 (15)	0.0146 (15)	0.0039 (16)
C3	0.040 (2)	0.038 (2)	0.033 (2)	0.0027 (17)	0.0134 (17)	0.0087 (17)
C4	0.030 (2)	0.035 (2)	0.032 (2)	0.0030 (16)	0.0069 (16)	−0.0047 (17)
C6	0.030 (2)	0.033 (2)	0.035 (2)	−0.0056 (15)	0.0115 (16)	−0.0059 (17)
C7	0.033 (2)	0.0275 (19)	0.0244 (18)	−0.0029 (15)	0.0087 (15)	−0.0031 (15)
C8	0.0320 (19)	0.0228 (18)	0.0204 (17)	−0.0048 (14)	0.0105 (15)	−0.0034 (14)
C9	0.0305 (19)	0.0217 (18)	0.0248 (18)	−0.0018 (14)	0.0069 (14)	−0.0029 (14)
C10	0.043 (2)	0.028 (2)	0.0243 (19)	0.0028 (16)	0.0104 (16)	−0.0006 (15)
C11	0.038 (2)	0.0272 (19)	0.030 (2)	0.0044 (16)	0.0060 (16)	−0.0005 (16)
C12	0.032 (2)	0.0268 (19)	0.034 (2)	0.0023 (15)	0.0106 (16)	−0.0014 (16)
C13	0.036 (2)	0.0242 (18)	0.0250 (18)	0.0006 (15)	0.0132 (15)	−0.0037 (15)
C14	0.0318 (19)	0.0179 (17)	0.0206 (17)	−0.0004 (14)	0.0087 (14)	−0.0005 (14)
O1	0.0353 (15)	0.0582 (18)	0.0454 (16)	0.0162 (12)	0.0137 (13)	0.0099 (13)
N1	0.0327 (16)	0.0248 (15)	0.0238 (15)	−0.0002 (12)	0.0085 (12)	−0.0020 (12)
N2	0.0335 (16)	0.0241 (15)	0.0207 (14)	0.0028 (12)	0.0089 (12)	0.0042 (12)
C5	0.038 (3)	0.065 (5)	0.054 (4)	0.012 (3)	0.010 (3)	0.003 (4)
F1	0.026 (2)	0.084 (3)	0.111 (3)	0.0205 (17)	0.021 (2)	0.043 (2)
F2	0.056 (2)	0.154 (5)	0.086 (3)	0.030 (3)	0.034 (2)	0.078 (3)
F3	0.076 (3)	0.085 (3)	0.116 (4)	0.016 (3)	−0.045 (3)	−0.042 (3)

### Geometric parameters (Å, º)

**Table:**

C1—C2	1.390 (4)	C11—C12	1.397 (5)
C1—C7	1.393 (4)	C11—H11	0.9300
C1—C8	1.469 (4)	C12—C13	1.380 (4)
C2—C3	1.397 (4)	C12—H12	0.9300
C2—H2	0.9300	C13—C14	1.380 (4)
C3—C4	1.376 (5)	C13—H13	0.9300
C3—H3	0.9300	C14—N2	1.385 (4)
C4—C6	1.382 (4)	O1—C5	1.332 (6)
C4—O1	1.417 (4)	N2—H21	0.8600
C6—C7	1.391 (4)	C5—F1	1.309 (5)
C6—H6	0.9300	C5—F3	1.321 (7)
C7—H7	0.9300	C5—F2	1.329 (8)
C8—N1	1.334 (4)	C51—F11	1.309 (5)
C8—N2	1.358 (4)	C51—F31	1.321 (7)
C9—N1	1.399 (4)	C51—F21	1.329 (8)
C9—C14	1.400 (4)	C52—F12	1.309 (5)
C9—C10	1.403 (4)	C52—F32	1.321 (7)
C10—C11	1.379 (5)	C52—F22	1.329 (8)
C10—H10	0.9300		
			
C2—C1—C7	119.5 (3)	C10—C11—H11	118.9
C2—C1—C8	120.0 (3)	C12—C11—H11	118.9
C7—C1—C8	120.4 (3)	C13—C12—C11	121.0 (3)
C1—C2—C3	120.5 (3)	C13—C12—H12	119.5
C1—C2—H2	119.8	C11—C12—H12	119.5
C3—C2—H2	119.8	C14—C13—C12	117.2 (3)
C4—C3—C2	118.6 (3)	C14—C13—H13	121.4
C4—C3—H3	120.7	C12—C13—H13	121.4
C2—C3—H3	120.7	C13—C14—N2	132.6 (3)
C3—C4—C6	122.1 (3)	C13—C14—C9	122.6 (3)
C3—C4—O1	120.8 (3)	N2—C14—C9	104.8 (3)
C6—C4—O1	116.9 (3)	C5—O1—C4	117.5 (3)
C4—C6—C7	118.7 (3)	C8—N1—C9	104.3 (3)
C4—C6—H6	120.6	C8—N2—C14	107.8 (3)
C7—C6—H6	120.6	C8—N2—H21	126.1
C6—C7—C1	120.4 (3)	C14—N2—H21	126.1
C6—C7—H7	119.8	F1—C5—F3	109.3 (5)
C1—C7—H7	119.8	F1—C5—F2	107.1 (5)
N1—C8—N2	112.7 (3)	F3—C5—F2	106.3 (6)
N1—C8—C1	124.7 (3)	F1—C5—O1	107.6 (5)
N2—C8—C1	122.5 (3)	F3—C5—O1	112.8 (5)
N1—C9—C14	110.4 (3)	F2—C5—O1	113.6 (5)
N1—C9—C10	129.7 (3)	F11—C51—F31	109.3 (5)
C14—C9—C10	119.9 (3)	F11—C51—F21	107.1 (5)
C11—C10—C9	117.1 (3)	F31—C51—F21	106.3 (6)
C11—C10—H10	121.4	F12—C52—F32	109.3 (5)
C9—C10—H10	121.4	F12—C52—F22	107.1 (5)
C10—C11—C12	122.2 (3)	F32—C52—F22	106.3 (6)

### Hydrogen-bond geometry (Å, º)

**Table:**

*D*—H···*A*	*D*—H	H···*A*	*D*···*A*	*D*—H···*A*
N2—H21···N1^i^	0.86	2.07	2.864 (4)	154

Symmetry code: (i) *x*, −*y*+3/2, *z*−1/2.
